# Naturalistic Emotion Decoding From Facial Action Sets

**DOI:** 10.3389/fpsyg.2018.02678

**Published:** 2019-01-18

**Authors:** Sylwia Hyniewska, Wataru Sato, Susanne Kaiser, Catherine Pelachaud

**Affiliations:** ^1^Kokoro Research Center, Kyoto University, Kyoto, Japan; ^2^Swiss Center for Affective Sciences, University of Geneva, Geneva, Switzerland; ^3^Human Behaviour Analysis Laboratory, Department of Psychology, University of Geneva, Geneva, Switzerland; ^4^Institut des Systèmes Intelligents et de Robotique (ISIR), Université Pierre et Marie Curie/Centre National de la Recherche Scientifique (CNRS), Paris, France

**Keywords:** emotional facial expression, spontaneous expressions, naturalistic, cognitive appraisal, nonverbal behavior

## Abstract

Researchers have theoretically proposed that humans decode other individuals' emotions or elementary cognitive appraisals from particular sets of facial action units (AUs). However, only a few empirical studies have systematically tested the relationships between the decoding of emotions/appraisals and sets of AUs, and the results are mixed. Furthermore, the previous studies relied on facial expressions of actors and no study used spontaneous and dynamic facial expressions in naturalistic settings. We investigated this issue using video recordings of facial expressions filmed unobtrusively in a real-life emotional situation, specifically loss of luggage at an airport. The AUs observed in the videos were annotated using the Facial Action Coding System. Male participants (*n* = 98) were asked to decode emotions (e.g., anger) and appraisals (e.g., suddenness) from facial expressions. We explored the relationships between the emotion/appraisal decoding and AUs using stepwise multiple regression analyses. The results revealed that all the rated emotions and appraisals were associated with sets of AUs. The profiles of regression equations showed AUs both consistent and inconsistent with those in theoretical proposals. The results suggest that (1) the decoding of emotions and appraisals in facial expressions is implemented by the perception of set of AUs, and (2) the profiles of such AU sets could be different from previous theories.

## Introduction

Reading emotions of other individuals from their facial expressions is an important skill in managing our social relationships. Researchers have postulated that emotional categories (e.g., anger) (Ekman and Friesen, 1978, 1982) or elementary components of emotions, such as cognitive appraisals (e.g., suddenness) (Scherer, 1984; Smith and Scott, 1997), can be decoded based on the recognition of specific sets of facial movements (Tables A1, A2 in Supplementary Material). For example, Ekman and Friesen (1978) proposed that specific sets of facial action units (AUs), which could be coded through the Facial Action Coding System (FACS; Ekman et al., 2002), could signal particular emotions and specified the required action unit sets. For instance, in the case of sadness, the facial action set includes inner eyebrows raised (AU 1) and drawn together (AU 4), and lip corners pulled down (AU 15) (Ekman and Friesen, 1975). Scherer (1984), on the other hand, proposed that sets of AUs could signal cognitive appraisals. These researchers developed their theories based on previous theories and findings and their intuitions (Ekman, 2005).

However, only a few previous empirical studies systematically investigated the theoretical predictions on the relationships between the decoding of emotional categories or cognitive appraisals and AU sets and these studies did not provide clear supportive evidence (Galati et al., 1997; Kohler et al., 2004; Fiorentini et al., 2012; Mehu et al., 2012). For example, Kohler et al. (2004) investigated how participants categorize four emotions expressed by actors (Kohler et al., 2004). Based on the results from their decoding study, the authors described the necessary facial AUs for recognizing emotional expressions of high intensity happy, sad, angry, and fearful faces. The analysis showed that the four emotions could be identified with sets of AUs specific to these emotions which are characteristic of the target emotions and distinct from the other three analyzed emotions. However, the profiles of AUs were only partially consistent with theoretical predictions. For example, the brow lowerer (AU4) was associated with the decoding of sadness, however the inner eyebrows raiser (AU 1) and lip corner depressor (AU 15) were not. In short, these studies showed that decoding of emotions and appraisals in facial expressions was associated with sets of AUs, but the profiles of AU sets were only partially consistent with the theoretical predictions.

Furthermore, it must be noted that none of the aforementioned studies evaluated spontaneous emotional expressions in naturalistic settings. Facial displays encountered in everyday life situations show high variability including blends between emotions (Scherer and Ellgring, 2007; Calvo and Nummenmaa, 2015), and spontaneous behavior is more ambiguous (e.g., Yik et al., 1998). This issue is particularly important as behaviors we see in real-life emotional situations are often not the prototypical ones described in literature– they are very varied in terms of co-existing facial movements and sometimes subtle, with rare and low-intensity facial actions (e.g., see Hess and Kleck, 1994; Russell and Fernández-Dols, 1997).

In this study, we investigated whether and how sets of facial actions could be associated with the decoding of emotions and appraisals in spontaneous facial expressions in a naturalistic setting. As stimuli of such spontaneous facial expressions, we used unobtrusive recordings from a hidden camera showing face-to-face interactions of passengers claiming the loss of their luggage at an airport (Scherer and Ceschi, 1997, 2000). All the AUs in the passengers' facial expressions were first coded using FACS (Ekman et al., 2002). We then asked participants to rate six emotions—two positive (Joy, Relief) and four negative (Anger, Sadness, Contempt, Shame)—as well as six appraisals: suddenness, goal obstruction, importance and relevance, coping potential, external norm violation, and internal norm violation. Surprise was not included given that some previous studies (Kohler et al., 2004; Mehu et al., 2012) showed no agreement regarding its valence (Fontaine et al., 2007; Reisenzein and Meyer, 2009; Reisenzein et al., 2012; Topolinski and Strack, 2015), and described its duration as shorter than that of other emotions, making it an affect that could be potentially of a different nature than the other studied emotions (Reisenzein et al., 2012). We explored the relationships between the emotion/appraisal decoding and facial actions using stepwise multiple regression analyses. We expected to observe that sets of facial actions enable the decoding of emotions and appraisals (Ekman, 1992; Scherer and Ellgring, 2007). We did not formulate predictions for the AUs expected in each set given the lack of former decoding studies focusing on data from naturalistic settings.

## Materials and Methods

### Participants

One hundred and twenty-two students from a French technical university took part in the study. A psychologist conducted a short interview with the participants and found that women, a minority in this technical school, were a non-homogenous population (great age distribution, reported psychological history, and intake of substances). Therefore, only data from male students were considered in the analysis (*n* = 98; age = 17–25, means ± *SD* = 19.0 ± 1.5). The interview did not lead to the detection of any neuropsychiatric or psychological history in any of the participants. All participants provided written informed consent prior to participation in the study and were debriefed after the study. The study was approved by the University of Geneva ethics committee and conducted in accordance with the approved guidelines.

### Stimuli

Our data relies on unobtrusive recordings from a hidden camera showing face-to-face interactions of passengers claiming the loss of their luggage at an airport (Scherer and Ceschi, 1997, 2000). The aim of such a naturalistic corpus was to obtain dynamic non-acted expressions, including non-typical and subtle facial displays.

**Figure 1:**
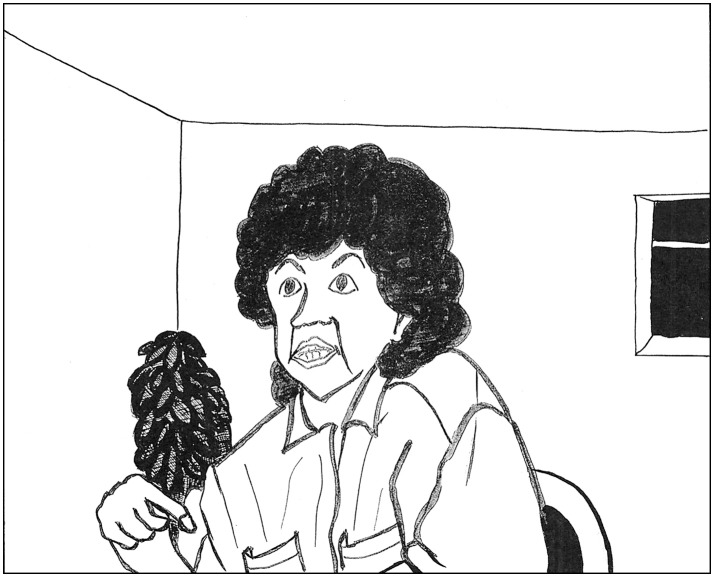
Schematic illustration of stimuli: frame presenting a passenger claiming the loss of luggage to a hostess. Actual stimuli were real-life dynamic videos.

Videos from this Lost Luggage corpus focus on the passenger, with a head and torso framing, while showing in the right corner a reduced size video of the face of the hostess (see Figure 1 for a schematic representation of stimuli).

The original corpus included 1 min long video clips (16-bit colors) that have not been cut in a way to depict only one mental state per segment, therefore the first task was to segment emotional extracts, i.e., define when an emotional state starts and when it ends.

We asked laypersons to watch and to mark in time all mental states and point out state changes. The task was explained through guidelines that were provided in a written format that was additionally read orally to make sure the participants thought carefully about all the provided examples.

The judges were told that their task was to indicate changes between different mental states of one person and that each mental state could be made of several affects happening at the same time, e.g., one mental state composed of 50% joy and 50% guilt. They had to select a period of time (by indicating a starting and an ending time) for each state and to define this mental state. To avoid guiding participants into a particular theoretical framework, guidelines provided examples of action tendencies, motivational changes, appraisal attributions and emotional labels. Judges were told orally that the focus is on “internal states” of passengers that have lost their luggage and that the films come from a hidden camera at an airport. Judges were told that in one video clip a passenger can display several mental states and moments of neutrality and that they had to indicate them all. They could describe what they see in sentences, through expressions or labels either orally (transcribed by the experimenter) or in a written format on a piece of paper or directly in the provided ANVIL software, with which they were assisted (ANVIL, Video Annotation Research Tool. http://www.dfki.de/kipp/anvil/).

Seven laypeople, administrative staff from the technical university, were invited to act as judges for the task. The two first judges to participate (an account officer and a junior secretary) found the task to be extremely difficult. They gave the following reasons:

- it is impossible to say that a state is changing;
- it could be possible to point that there is an emotion such as anger in a video, but not point to a time;
- defining what the passengers feel or in what state they are, without being guided by specific emotional labels is difficult.

A third judge reported that the observed passengers are talking and not experiencing any affects or changes in mental states and therefore it is impossible to fulfill the task.

Consequently we decided to assign this procedure to individuals who we expected to have some ease to fulfill the task: e.g., individuals who have developed some acuity in the perception of facial expressions. Three individuals were recruited according to their professional activity (virtual character synthesis; facial graphics; FACS coding) and one for his interest in the non-verbal communication and social cognition. All four individuals, that we called “expert judges,” understood the task straight away from reading the guidelines.

Each clip was annotated by three expert judges.

In case of ambiguity, for example when one expert out of three considered less changes in a clip than the other experts, and made a segment last longer, we opted for leaving out the non-agreed upon segment. To reformulate, the solution was, when possible, to recut the clip to eliminate moments that led to discordance. Only moments on which judges agreed to display only one state were kept. If a state starting during a movement or a sentence was preceded by a neutral phase, a second or a second and a half might have been added to the chosen segment to enable the display of the movement development.

In two cases in which ambiguity did not allow an easy and straightforward cutting even in the above, restrictive, manner, a fourth experienced judge was asked to annotate the video clips. In both cases, two judges annotated long segments and one judge a much shorter segment. The fourth judge had a very similar segmentation to the short segmentation, for the two concerned videos. Thus, we followed this restrictive segmentation, as it enabled a definition of mental states to be extracted and presented in separate clips.

After cutting, 64 clips were obtained. Several extracts from these were excluded from the corpus, as they involved a fragment where the face was majorly obstructed or hidden behind glasses that reflected light in the view of the camera, or were presenting a situation outside of the original canvas (e.g., talking to a third person). In the end, 39 clips were included in the study, each lasting 4–56 s, with a majority lasting between 20 and 28 s. The clips were encoded with a temporal resolution up to 1/25th of a second and showed 19 male and 20 female stimuli. The passengers presented in the clips came from a wide variety of cultural backgrounds. Preliminary analyses showed that there were no effects of gender of stimuli in terms of all of the AU and emotion/appraisal rating data (*t*-test, *p* > 0.1); accordingly, the factor of stimulus gender was omitted in the following analyses.

### FACS Coding

As we wanted to associate short video extracts to attributions made by laypersons, it was important to code all the facial actions that could have an impact on the observers. The ANVIL software was used for the annotation, with 61 tracks for the face (FACS; Ekman et al., 2002) and 22 for the bodily action coding in time. The analysis of the latter coding is outside the scope of this article. The FACS coding was performed by a certified FACS coder and was verified by a second certified FACS coder. The second coder annotated 12 % of the videos (randomly assigned). Both coders used the FACS manual as a constant reference criterion.

In assessing the precision of scoring, we looked at the frame-by-frame agreement by computing Cohen's Kappa (*k*) for face action coding (Cohen, 1960). The mean agreement was observed at the *k* = 0.66 (*SD* = 0.18), which according to Cicchetti and Sparrow (1981) shows strong agreement. Each of our particular AU coding cases showed satisfactory agreement except for AU 20 (lip stretcher), where *k* in the 0.21–0.40 range indicated merely a weak/fair agreement.

### Procedure

Participants arrived in groups of two to ten. Each participant accessed the study individually through a web browser. The guidelines provided on the first web page were sufficient for understanding the tasks. Participants were randomly attributed to rating blocks. Emotional labels were presented in two controlled orders, the same order of presentation being kept for all stimuli judged by the same participant. Participants watched and evaluated from 6 to 39 short video clip extracts, depending on their self-reported concentration level and their willingness to participate. They answered the same set of questions after each video.

On the first page after each video display, participants were asked to evaluate appraisals presented in the form of a sentence, such as “Do you have the impression that the person you saw in the video, just faced a sudden event?” (suddenness). Appraisals were presented in the chronological order defined by the Componential theory: suddenness, goal obstruction, important and incongruent event, coping potential, respect of internal standards, and violation of external standards (e.g., Scherer, 2001). Participants answered appraisal questions on a 7-point Likert scale, ranging from 0 = totally disagree to 6 = totally agree.

On the second page after the video, participants also had to judge whether the observed passenger was experiencing joy, anger, relief, sadness, contempt, fear and shame. Each emotion was evaluated by participants on a separate 7-point Likert scale ranging from zero (no emotion) to six (strong emotion) and the emotions were not mutually exclusive. The order of presentation of emotional labels was randomized. The mean attribution of each label to each video (across participants) was the dependent variable. The independent variable was the duration of the facial action cues annotated by coders as present in videos watched by participants.

### Data Analysis

For each video (*n* = 39), the annotation in terms of FACS units was quantified by computing the total duration of this AU in a video. We selected this measure as the length of videos was dependent on the duration of present AUs leading to the decoding of a mental state, and therefore the percentage of time an action is present in a video clip is not informative. Stepwise regression analyses with backward selection were performed using SPSS 16.0J (SPSS Japan, Tokyo, Japan). Stepwise regression analyses are techniques for selecting a subset of predictor variables (Ruengvirayudh and Brooks, 2016). By conducting the analyses, we tested whether and how the subset of AUs could predict the decoding of specific emotions/appraisals. Individual regression analyses were conducted for each emotion/appraisal as the dependent variable. All AUs were first entered into the model as independent variables and AUs that did not significantly predict the dependent variable were removed from the model one by one. The first model for which all AUs helped predict at least a marginally significant (*p* < 0.10) variance in the dependent variable emotion/appraisal was selected as the final model. Before the analyses, we conducted *a priori* power analyses using G^*^Power 3.1.9.2 (Faul et al., 2007). We used the data of Galati et al. (1997) as prior information, because only this study applied similar regression approaches and reported sufficient information for power analyses. The number of AUs associated with emotion decoding were comparable across previous studies (mean, 6.0, 5.25, and 7.1 in Galati et al., 1997; Kohler et al., 2004; and Fiorentini et al., 2012, respectively). The results showed that our regression analyses could detect the relationships between the AU sets and decoding of emotional categories reported in (Galati et al., 1997). (mean *R*^2^ = 0.49) with a strong statistical power (α = 0.05; 1–β = 0.99). Based on these data, we expected that our variable selection approach using stepwise regression analyses could detect the set of AUs similar with previous studies in terms of size. However, our analyses lacked the power to investigate full or larger sets of AUs (see discussion). For the final models, we calculated squared multiple correlation coefficients (*R*^2^) as effect-size parameters. Also, we calculated *post hoc* statistical power (1–β) for *R*^2^ deviation from zero using G^*^Power 3.1.9.2 (Faul et al., 2007).

## Results

The FACS coding (total duration of AUs) and means ± *SD*s of attribution ratings are shown in Tables 1, 2, respectively.

**Table 1:** Total duration of action units (AUs) observed in each video.

**Videos**	**Action unit**																													
	**1**	**2**	**4**	**5**	**6**	**7**	**8**	**10**	**11**	**12**	**13**	**14**	**15**	**16**	**17**	**18**	**20**	**22**	**23**	**24**	**25**	**26**	**27**	**28**	**29**	**32**	**34**	**37**	**43**	**50**
	**Inner brow raiser**	**Outer brow raiser**	**Brow lower**	**Upper lid raiser**	**Cheek raiser**	**Lid tighten**	**Lips toward each other**	**Upper lip raiser**	**Nasolabial deepener**	**Lip corner puller**	**Sharp lip puller**	**Dimpler**	**Lip corner depressor**	**Lower lip depressor**	**Chin raiser**	**Lip puckerer**	**Lip stretch**	**Lip funnel**	**Lip tighten**	**Lip pressor**	**Lips part**	**Jaw drop**	**Mouth stretch**	**Lip suck**	**Jaw thrust**	**Lip bite**	**Cheek puff**	**Lip wipe**	**Eyes closed**	**Speech**
1	6.1	3.1	0.0	0.8	0.0	0.2	0.0	3.7	0.0	0.2	0.0	0.0	0.0	0.0	0.0	0.0	0.0	0.0	0.0	0.0	6.4	9.3	0.0	0.0	0.0	0.0	0.0	0.0	2.6	3.8
2	24.0	1.2	23.3	0.0	0.0	4.9	0.0	0.0	4.9	0.0	0.0	0.0	0.0	0.0	2.4	0.0	2.0	0.0	2.0	0.2	2.6	0.0	0.0	0.0	0.9	0.0	0.0	0.0	0.0	22.8
3	0.8	5.1	1.0	0.8	0.0	1.8	0.0	1.2	0.0	0.0	0.0	1.6	0.0	0.0	0.5	0.0	4.2	0.0	0.6	0.4	5.0	2.8	1.3	0.0	1.7	0.0	0.0	0.0	0.0	11.2
4	1.7	1.3	0.5	0.0	0.0	0.8	0.0	2.1	0.0	0.3	0.0	0.1	1.5	0.0	0.0	0.0	0.0	0.0	1.5	0.3	0.0	0.0	0.0	0.0	0.0	0.0	0.0	0.0	0.3	0.0
5	0.0	0.0	0.0	0.0	0.0	3.7	0.0	3.0	0.6	2.2	0.0	0.0	0.0	0.0	1.1	0.0	0.9	0.0	0.0	0.0	0.0	0.0	0.0	0.0	0.0	0.0	0.0	0.0	0.0	6.7
6	0.0	0.0	0.0	0.0	0.0	1.1	0.0	1.5	0.0	1.8	0.0	0.1	0.6	0.2	1.0	0.0	0.4	0.0	1.6	0.0	0.0	0.0	0.0	0.0	0.0	0.0	0.0	0.0	0.0	14.7
7	0.0	5.4	3.0	0.0	0.0	3.3	0.0	0.0	0.0	0.0	0.0	0.7	0.3	0.0	0.5	0.0	1.0	0.0	1.7	2.3	2.7	3.2	0.0	0.0	0.0	0.0	0.2	0.0	0.0	7.1
8	2.4	5.2	0.0	1.6	0.5	2.6	0.0	0.0	0.0	0.0	0.0	0.0	0.4	0.2	1.8	0.0	0.0	0.0	5.1	4.6	1.0	0.4	0.0	0.0	0.0	0.0	0.0	0.0	0.0	1.9
9	0.0	0.0	0.0	0.0	0.0	12.4	0.0	0.0	0.0	0.0	0.0	0.0	0.0	0.0	3.8	2.5	0.0	0.0	1.8	0.8	0.0	0.0	0.0	0.0	0.0	0.0	0.0	0.0	0.0	0.0
10	1.4	0.7	1.7	0.0	0.0	0.0	0.0	4.0	0.0	0.0	0.0	0.5	2.1	0.0	6.0	0.0	0.0	0.0	0.0	0.0	2.3	1.0	1.2	0.0	0.0	0.0	0.0	0.0	1.1	0.0
11	9.2	9.2	9.1	0.0	0.0	7.6	0.0	8.5	0.0	0.0	0.0	5.8	0.6	0.0	9.0	0.0	1.6	0.0	0.0	4.5	0.0	0.0	0.0	0.0	0.0	0.0	0.0	0.0	0.0	2.9
12	9.5	8.7	9.4	0.0	0.0	6.4	0.0	6.2	0.5	0.0	0.0	0.0	0.0	0.0	0.5	0.1	0.0	0.0	0.0	0.5	4.1	8.0	0.0	0.0	0.0	0.0	0.0	0.0	0.5	3.3
13	1.4	3.1	8.9	0.0	0.0	0.0	0.0	0.0	0.0	0.0	0.0	0.0	0.1	0.0	2.8	0.0	0.0	0.0	0.0	0.1	0.0	0.0	0.0	0.0	0.0	0.0	0.0	0.0	0.0	0.0
14	7.4	3.6	6.9	0.0	0.0	0.0	0.0	0.4	0.0	0.0	0.0	0.7	0.4	3.4	0.5	0.0	1.2	0.0	0.2	0.0	12.2	12.8	0.0	0.0	0.0	0.0	0.0	0.0	0.0	3.6
15	9.7	2.3	10.8	0.0	0.0	0.0	0.0	0.0	0.0	0.0	0.0	0.0	0.4	14.6	0.5	0.0	0.8	0.0	0.8	0.0	15.1	2.3	0.0	0.0	0.0	0.0	0.0	0.0	0.0	0.3
16	0.0	0.0	0.0	0.0	0.0	0.0	0.0	4.0	0.0	3.1	0.3	0.2	0.0	0.0	0.6	0.0	0.0	0.0	0.8	0.0	0.4	0.0	0.0	0.0	0.0	0.0	0.0	0.0	0.0	8.4
17	0.0	0.0	0.0	0.0	0.0	3.9	0.0	0.0	0.0	0.7	0.0	0.2	0.0	0.0	0.0	0.0	0.0	0.0	1.8	0.2	4.0	4.0	0.0	0.0	0.0	0.0	0.0	0.0	0.0	0.0
18	0.0	0.0	0.0	0.0	0.0	0.0	0.0	0.0	0.0	0.0	0.0	0.0	0.0	0.0	0.0	0.0	0.0	0.0	0.0	0.0	0.7	0.7	0.0	0.0	0.0	0.0	0.0	0.0	0.0	0.0
19	6.8	5.8	1.3	1.8	0.0	1.4	0.0	0.0	0.0	0.0	0.0	0.3	0.0	0.0	7.8	0.0	0.0	0.0	0.0	0.0	0.8	0.0	0.0	0.0	0.0	0.0	0.0	0.2	0.0	9.9
20	8.6	11.0	8.9	3.9	0.0	0.0	0.0	0.0	0.0	0.0	0.0	0.3	0.0	0.0	0.0	0.0	0.0	0.0	0.0	0.0	2.6	2.8	0.0	0.0	0.0	0.0	0.0	0.0	0.0	0.0
21	10.5	0.0	0.0	0.0	0.0	12.1	0.0	0.0	0.0	11.0	0.0	1.0	0.0	0.0	0.0	0.0	0.0	0.0	10.5	0.0	0.0	0.0	0.0	0.0	0.0	0.0	0.0	0.0	0.0	18.5
22	0.8	8.3	0.0	1.1	5.4	0.9	0.0	1.8	0.0	0.4	0.0	1.3	2.1	0.0	2.8	0.0	0.0	0.0	0.0	0.0	1.7	4.6	0.0	0.0	0.0	0.0	0.0	0.0	0.7	3.5
23	4.0	0.0	2.7	0.0	0.0	0.0	0.0	0.0	0.0	0.0	0.0	2.2	1.6	0.0	3.4	0.0	0.6	0.0	0.0	0.4	0.0	0.0	0.0	0.0	0.0	0.0	0.0	0.0	0.0	1.7
24	2.7	2.6	7.4	0.3	0.0	3.1	0.0	2.9	0.0	0.0	0.0	0.0	0.0	0.0	0.0	0.0	0.0	0.0	0.0	0.0	6.9	6.4	0.0	0.0	1.8	0.0	0.0	0.0	0.0	8.3
25	3.0	3.2	0.2	0.0	0.0	0.1	0.0	1.6	0.0	0.0	0.0	3.4	0.0	0.0	0.2	0.0	0.1	0.0	1.0	0.0	1.6	1.5	0.0	0.0	0.0	0.0	0.0	0.0	0.0	0.0
26	3.8	2.5	1.8	3.5	0.0	3.3	0.0	0.0	0.0	0.0	0.0	2.2	0.0	0.0	0.0	0.0	0.0	0.0	0.0	0.0	0.4	0.4	0.0	0.0	0.0	0.0	0.0	0.0	0.0	0.8
27	0.7	2.0	2.3	0.9	0.0	2.6	0.0	0.0	0.0	0.0	0.0	2.0	0.0	0.0	0.0	0.0	0.0	0.0	0.0	0.0	0.0	0.7	0.0	0.0	0.0	0.0	0.0	0.0	0.1	0.0
28	7.5	0.0	0.5	0.0	0.0	0.4	0.0	0.0	0.0	0.0	0.0	0.0	0.0	0.0	0.0	0.0	0.0	0.0	0.0	0.0	0.0	0.0	0.0	0.0	0.0	0.0	0.0	0.0	0.0	0.0
29	6.9	0.0	0.0	0.0	0.0	0.0	0.0	0.0	0.2	0.0	0.0	0.0	0.0	0.0	0.0	0.0	0.0	0.0	0.0	0.0	8.3	0.0	0.0	0.0	0.0	0.0	0.0	0.0	0.0	0.0
30	0.2	0.2	0.0	0.0	0.2	0.0	0.0	0.0	0.0	2.6	1.1	0.9	0.0	0.0	1.8	0.0	0.0	0.1	0.9	2.3	0.9	0.0	0.0	0.0	0.0	0.0	0.0	0.0	0.0	0.0
31	7.0	0.6	1.9	0.0	0.0	0.4	0.0	0.0	0.0	0.0	0.0	17.1	0.0	0.0	3.3	0.0	0.0	0.0	0.0	0.4	1.8	2.1	0.0	0.0	0.0	0.0	0.0	0.0	0.0	0.0
32	7.0	0.6	1.9	0.0	0.0	0.4	0.0	0.0	0.0	0.0	0.0	17.1	0.0	0.0	3.3	0.0	0.0	0.0	0.0	0.4	1.8	2.1	0.0	0.0	0.0	0.0	0.0	0.0	0.0	0.0
33	0.0	0.6	0.0	1.2	0.0	0.0	1.2	0.0	0.0	0.0	0.0	0.0	0.0	0.0	0.0	0.0	0.2	0.0	0.0	0.0	4.1	4.0	0.0	0.0	0.0	0.0	0.0	0.0	0.0	2.2
34	3.2	3.5	5.2	0.9	0.7	4.5	0.0	2.0	0.0	1.7	0.0	0.5	0.0	0.0	0.0	0.0	0.0	0.2	0.2	0.0	2.8	1.2	0.0	0.0	0.0	0.0	0.0	0.0	0.0	2.6
35	10.4	11.1	0.0	0.0	2.1	11.4	0.0	3.1	0.0	5.7	0.0	1.8	0.0	0.0	0.3	0.0	0.0	0.0	0.2	0.0	3.3	3.4	0.0	0.0	0.0	0.0	0.0	0.0	0.0	7.2
36	1.7	1.8	0.8	0.0	0.0	0.9	0.0	0.2	0.0	0.0	0.0	0.6	0.0	0.0	0.4	0.0	0.0	0.0	0.0	0.0	0.0	2.4	0.0	0.0	0.0	0.0	0.0	0.0	0.0	8.9
37	1.7	4.9	1.0	0.0	0.0	5.9	0.0	1.8	0.2	1.2	0.0	0.0	1.1	0.0	0.9	0.0	0.4	0.0	0.0	0.0	2.2	2.5	0.0	0.0	2.3	0.0	0.0	0.0	0.7	2.9
38	16.8	8.0	7.5	0.0	0.0	21.9	0.0	0.0	0.0	0.0	0.0	8.3	0.0	0.0	5.0	0.0	0.2	0.0	14.9	5.0	2.2	3.7	0.0	7.5	0.0	1.0	0.0	0.0	0.0	26.4
39	0.0	0.0	0.0	0.0	0.0	14.3	1.7	0.0	0.0	0.0	0.0	1.4	0.0	0.0	5.9	0.0	0.0	0.0	8.6	0.0	0.0	0.0	0.0	2.4	1.0	0.0	0.0	0.0	0.0	11.6

*Only action units with >0 durations are reported*.

**Table 2:** Means ± standard deviations of participants' ratings for each video in terms of emotion and appraisal label attributions.

**Videos**	**Attributions**												
	**Joy**	**Anger**	**Relief**	**Sadness**	**Contempt**	**Fear**	**Shame**	**Suddenness**	**Goal Obstructive**	**Important and relevant**	**Coping potential**	**External norm violation**	**Respect of internal standards**
1	0.18 (0.5)	0.41 (0.8)	1.18 (1.44)	1.36 (1.43)	0.45 (0.86)	1.5 (1.47)	0.73 (1.08)	4.27 (1.49)	3.95 (1.33)	3.91 (1.66)	3.95 (1.79)	3 (1.41)	5.45 (1.06)
2	0.13 (0.34)	1.06 (1)	0.63 (0.89)	2 (1.63)	1.69 (1.82)	2.06 (1.98)	0.63 (0.96)	5.38 (1.59)	5.38 (1.59)	4.94 (1.69)	4.38 (1.78)	3.25 (1.57)	4.94 (1.61)
3	0.29 (0.59)	0.29 (0.47)	1.71 (1.21)	0.29 (0.47)	0.76 (1.3)	0.82 (1.29)	0.47 (0.72)	4.24 (1.64)	3.35 (1.62)	3.41 (1.5)	5.65 (1.17)	3.29 (1.49)	5.71 (1.1)
4	0.5 (0.94)	1.57 (1.91)	0.57 (1.02)	0.36 (0.74)	2.57 (1.95)	0.5 (1.09)	0.21 (0.58)	0.07 (0.27)	1.57 (1.7)	0.29 (0.47)	2 (2.11)	1.36 (2.02)	2.57 (1.99)
5	0.35 (0.86)	0.47 (0.72)	0.88 (1.17)	0.82 (0.81)	0.29 (0.77)	0.71 (0.77)	0.53 (0.72)	3.65 (1.8)	3.94 (1.95)	4.06 (1.52)	4.59 (1.37)	3.65 (1.62)	5.06 (1.14)
6	0.47 (0.9)	0.47 (0.7)	0.84 (1.34)	0.68 (1)	0.68 (0.82)	0.74 (0.87)	0.32 (0.67)	4.47 (1.71)	3.95 (1.58)	3.89 (1.52)	5.47 (1.22)	3.26 (1.52)	5.63 (1.21)
7	0.21 (0.58)	1.86 (2.18)	0.57 (0.94)	1.21 (1.48)	1.86 (1.75)	1.5 (1.79)	1.07 (1.98)	3.43 (1.65)	3.93 (1.94)	3.64 (1.82)	4.57 (1.7)	3.14 (1.46)	5 (1.52)
8	0.04 (0.19)	1.57 (1.73)	0.31 (0.67)	1.66 (1.78)	1.43 (1.79)	2.06 (1.82)	0.92 (1.16)	4.53 (1.87)	4.51 (1.78)	4.25 (1.7)	3.7 (1.77)	3.99 (1.7)	4.69 (1.32)
9	0.03 (0.16)	1.3 (1.32)	0.13 (0.4)	2.18 (1.84)	1.03 (1.42)	2.08 (2)	1 (1.28)	4.9 (1.61)	4.95 (1.54)	4.98 (1.51)	3.15 (1.55)	3.65 (1.42)	5.23 (1.17)
10	0.16 (0.5)	2.74 (2)	0.05 (0.23)	1.37 (1.71)	2.74 (2.16)	1.42 (1.89)	0.74 (0.93)	4.74 (1.73)	5.26 (1.52)	4.53 (1.68)	3.32 (1.7)	4.95 (1.72)	4.32 (1.86)
11	0 (0)	2.32 (2.38)	0 (0)	0.89 (1.49)	1.89 (2.13)	0.53 (0.96)	0.84 (1.12)	3.95 (2.27)	3.84 (2.36)	3.74 (2.38)	3.21 (1.93)	3.79 (2.27)	3.37 (1.8)
12	0 (0)	1.65 (2.18)	0 (0)	0.65 (1)	0.94 (1.6)	0.41 (0.87)	1 (1.12)	2.88 (2.18)	3 (2.21)	3.12 (2.12)	2.65 (1.9)	2.71 (1.79)	2.88 (2.03)
13	0.03 (0.16)	1.69 (1.34)	0.15 (0.43)	1.26 (1.37)	1.79 (1.79)	1.54 (1.6)	0.54 (1.1)	4.44 (1.64)	4.69 (1.56)	4.41 (1.57)	3.49 (1.47)	3.97 (1.58)	4.44 (1.43)
14	0.1 (0.31)	1 (1.41)	0.6 (0.94)	1.45 (1.47)	0.7 (1.26)	2.15 (1.5)	0.3 (0.73)	5 (1.49)	4.7 (1.84)	4.35 (1.98)	4 (1.81)	3.05 (2.01)	5.4 (1.57)
15	0 (0)	0.95 (1.47)	0.11 (0.32)	2.26 (1.94)	0.37 (0.76)	3.37 (1.61)	0.26 (0.65)	5.68 (1.29)	5.58 (1.43)	5.53 (1.31)	2.63 (1.71)	3.63 (1.64)	4.42 (1.35)
16	0.53 (0.9)	2.63 (1.54)	0.05 (0.23)	0.37 (0.5)	2.37 (1.8)	0.89 (1.05)	0.63 (0.96)	5.26 (1.41)	5.21 (1.55)	5.11 (1.41)	4.63 (1.61)	5.79 (1.4)	5.47 (1.17)
17	0.27 (0.47)	0.55 (0.82)	0.55 (0.93)	0.64 (0.5)	0.27 (0.65)	1.55 (1.21)	0.27 (0.65)	5.27 (1.49)	4.73 (1.85)	5 (1.48)	3.91 (1.81)	3.73 (2.15)	4.45 (1.97)
18	0.11 (0.32)	1.89 (1.66)	0.42 (0.84)	0.74 (0.93)	1.21 (1.55)	1.11 (1.15)	1.47 (1.54)	3.79 (1.65)	4.26 (1.37)	3.84 (1.71)	5.05 (1.39)	4.74 (1.28)	4.95 (1.81)
19	0.14 (0.36)	0.79 (1.25)	0.36 (0.63)	1.93 (1.69)	0.86 (1.41)	2.5 (1.95)	0.86 (1.03)	2.64 (1.34)	6.14 (1.03)	2.93 (1.54)	4.29 (2.09)	4.64 (1.74)	4.21 (1.53)
20	0.14 (0.67)	1.79 (1.83)	0.11 (0.46)	1.99 (1.86)	1.78 (1.95)	2.86 (2.04)	0.89 (1.33)	4.85 (1.76)	4.95 (1.73)	4.71 (1.75)	2.6 (1.53)	3.97 (1.49)	4.11 (1.4)
21	0.24 (0.56)	1.12 (1.05)	0.88 (1.45)	0.53 (0.72)	1.47 (1.91)	1.06 (1.03)	1.06 (1.34)	4.65 (1.66)	4.12 (1.58)	4.18 (1.59)	5 (1.62)	4.06 (2.01)	4.94 (1.48)
22	0 (0)	1 (1.28)	0.08 (0.29)	1.75 (2.14)	1.17 (1.95)	2.58 (2.39)	1.5 (1.57)	5 (2.04)	4.83 (2.12)	4.75 (2.01)	2 (1.21)	3.25 (1.66)	3.92 (1.93)
23	1.29 (2.11)	1 (1.28)	1.37 (2)	0.9 (1.45)	1.1 (1.41)	2.17 (1.86)	1.68 (1.75)	4.15 (1.92)	4.83 (1.26)	3.46 (2.36)	3.02 (1.42)	4.02 (1.35)	4.56 (1.53)
24	0.03 (0.16)	1.5 (1.3)	0.3 (0.76)	0.98 (1.33)	1.9 (1.93)	0.83 (1.2)	1.03 (1.58)	3.65 (1.59)	3.83 (1.66)	3.7 (1.38)	5 (1.32)	3.58 (1.75)	4.6 (1.46)
25	0.68 (1.68)	2.4 (1.73)	0.51 (1.14)	2.05 (1.63)	2.21 (1.86)	1.92 (1.68)	0.77 (1.34)	4.6 (1.61)	5.3 (1.51)	4.12 (2.05)	2.84 (1.55)	4.48 (1.8)	3.6 (1.82)
26	0.2 (0.89)	0.8 (0.89)	0.15 (0.49)	1.75 (1.77)	0.75 (1.07)	2.55 (1.82)	1.9 (2.15)	5.75 (1.12)	5.1 (1.62)	4.95 (1.39)	3.1 (1.59)	3.3 (1.75)	4.05 (1.79)
27	0.07 (0.27)	1.57 (1.7)	0.29 (0.47)	2 (2.11)	1.36 (2.02)	2.57 (1.99)	1.36 (1.95)	5.14 (1.41)	5.36 (1.28)	5.21 (1.19)	3.07 (1.44)	3.29 (2.05)	4.64 (1.22)
28	0.2 (0.73)	2.31 (1.55)	0.15 (0.59)	3.05 (1.93)	1.6 (1.77)	2.22 (1.98)	1.69 (1.95)	3.95 (2.53)	4.13 (2.56)	4.49 (2.04)	2.27 (1.73)	3.4 (2.28)	4.16 (1.71)
29	0.03 (0.16)	1.75 (1.25)	0.92 (1.91)	3.32 (1.74)	2.08 (1.92)	2.1 (1.63)	0.86 (1.32)	4.82 (2.14)	5.16 (1.61)	4.9 (1.67)	2.64 (1.56)	4.3 (1.65)	4.29 (1.5)
30	1.63 (1.99)	2 (1.89)	0.88 (1.29)	1.15 (1.47)	1.21 (1.81)	1.67 (1.69)	1.9 (1.75)	3.52 (2.36)	3.75 (2.38)	4.56 (1.69)	3.56 (2.25)	4.06 (1.81)	4.83 (1.53)
31	0 (0)	2.03 (1.83)	0.13 (0.65)	1.23 (1.44)	1.6 (1.93)	1.4 (1.53)	1.4 (1.69)	4.72 (1.57)	4.9 (1.84)	4.68 (1.75)	3.38 (1.53)	4.3 (1.83)	4.43 (1.38)
32	1.21 (1.67)	1 (1.57)	1.64 (1.95)	0.86 (0.95)	0.86 (1.83)	0.79 (0.89)	0.64 (0.84)	4.64 (1.45)	4.14 (1.56)	3.79 (1.53)	4.79 (1.67)	3.57 (1.91)	5.21 (1.76)
33	0.26 (0.73)	0.68 (0.95)	0.63 (0.96)	0.37 (0.68)	0.68 (1.06)	0.95 (1.03)	0.42 (0.96)	4.37 (1.74)	3.68 (1.45)	4.21 (1.65)	4.16 (1.5)	3.58 (1.61)	3.95 (1.65)
34	0.63 (0.76)	1.32 (1.57)	0.95 (1.27)	0.47 (0.7)	1 (1.56)	0.89 (1.2)	0.63 (1.01)	4.74 (1.59)	4.42 (1.61)	4.37 (1.67)	3.84 (1.71)	3.63 (1.64)	4.16 (1.57)
35	2.82 (1.74)	0.24 (0.66)	1.82 (1.67)	0.24 (0.44)	0.24 (0.75)	0.12 (0.33)	0.59 (1.12)	3.41 (1.58)	3.06 (1.3)	3 (1.27)	5.41 (1.33)	2.47 (1.33)	5 (1.41)
36	0.99 (1.86)	1.44 (1.22)	1.18 (1.83)	0.64 (0.94)	1.29 (1.52)	1.12 (1.4)	0.65 (1.11)	3.61 (2.04)	4.21 (1.68)	3.49 (2.07)	5.05 (1.78)	3.74 (1.59)	5.25 (1.4)
37	0.41 (0.8)	1.94 (1.82)	0.47 (0.94)	1.59 (1.66)	0.94 (1.2)	1.59 (1.46)	0.88 (1.05)	3.53 (1.62)	4.71 (1.31)	5 (1.62)	4.88 (1.62)	4.06 (1.25)	0.94 (1.82)
38	0.18 (0.45)	0.63 (0.81)	0.73 (1.22)	1.43 (1.47)	0.65 (1.08)	1.28 (1.48)	0.9 (1.41)	4.48 (1.6)	4.2 (1.77)	4.55 (1.45)	4.68 (1.69)	3.1 (1.5)	5.48 (1.3)
39	0,42 (0,84)	0,16 (0,37)	1,37 (1,5)	0,58 (1,02)	0,21 (0,54)	0,53 (0,77)	0,37 (0,83)	4,58 (1,57)	3,84 (1,54)	4,32 (1,25)	4,74 (1,56)	2,95 (1,08)	5,21 (1,44)

Stepwise regression analyses with backward selection showed that the attributions of all emotional categories and cognitive appraisals were significantly predicted by sets of AUs (Table 3). All the final regression models showed high effect-size parameters (*R*^2^ > 0.46) and high statistical power (1–β > 0.99).

**Table 3:** Results of final models in stepwise regression analyses.

**Dependent variable**	***F***	***df***	***p***	***R*^**2**^**	**1 - β**
Joy	6.54	6.34	0.000	0.54	1.00
Anger	4.83	14.26	0.000	0.72	1.00
Sadness	5.00	10.30	0.000	0.63	1.00
Contempt	4.44	14.26	0.001	0.71	1.00
Fear	11.74	8.32	0.000	0.75	1.00
Shame	3.73	22.18	0.003	0.82	1.00
Suddenness	4.43	11.29	0.001	0.63	1.00
Goal obstruction	5.88	6.34	0.000	0.51	1.00
Relevance and discrepancy	3.57	10.30	0.003	0.54	1.00
Coping potential	4.39	16.24	0.001	0.75	1.00
External standards violation	3.56	8.32	0.005	0.47	1.00
Internal standards violation	4.74	18.22	0.000	0.80	1.00

When we evaluated the profiles of AUs predicting each emotion/appraisal (Table 4), we found that several predictions based on prior observations in the literature concerning the relation between facial actions and emotion/appraisal attributions were confirmed. Specifically, positive associations were found between joy and AU 12 (upward lip corner pulling); between anger and AU 1 (inner eyebrow raise) and AU 10 (nasolabial furrow deepening); between sadness and AU 1 (inner brow raise) between fear and AU 5 (opening of the eye/upper lid raise) and marginally AU 1 (inner brow raise); and between shame and AU 2 (outer brow raise), AU 5 (opening of the eye/upper lid raise), AU 20 (lip stretch), AU 25 (mouth opening) and marginally AU 7 (lower eyelid contraction). In terms of cognitive appraisals, goal obstruction and perception of an event as relevant but incongruent were positively associated with AU 17 (chin raise). Perception of coping potential was associated with AU 4 (brow lowering) and AU 24 (lip pressing).

**Table 4:** Results of significant independent variables of final models in stepwise regression analyses.

**Dependent variable**	**Statistics**	**Action unit**																													
		**1**	**2**	**4**	**5**	**6**	**7**	**8**	**10**	**11**	**12**	**13**	**14**	**15**	**16**	**17**	**18**	**20**	**22**	**23**	**24**	**25**	**26**	**27**	**28**	**29**	**32**	**34**	**37**	**43**	**50**
		**Inner brow raiser**	**Outer brow raiser**	**Brow lower**	**Upper lid raiser**	**Cheek raiser**	**Lid tighten**	**Lips toward each other**	**Upper lip raiser**	**Nasolabial deepener**	**Lip corner puller**	**Sharp lip puller**	**Dimpler**	**Lip corner depressor**	**Lower lip depressor**	**Chin raiser**	**Lip puckerer**	**Lip stretch**	**Lip funnel**	**Lip tighten**	**Lip pressor**	**Lips part**	**Jaw drop**	**Mouth stretch**	**Lip suck**	**Jaw thrust**	**Lip bite**	**Cheek puff**	**Lip wipe**	**Eyes closed**	**Speech**
Joy	β								−0.38		1.12									−1.70	0.53				2.42		−1.42				
	*t*								2.82		6.14									5.22	3.26				4.08		3.07				
	*p*								0.008		0.000									0.000	0.003				0.000		0.004				
Anger	β	0.58	−0.23				−0.98		0.65			0.28					0.28	−0.23		0.69		−0.34				0.41	0.30	0.38		−0.30	−0.80
	*t*	3.82	1.71				3.76		4.39			2.51					1.98	1.72		2.69		2.74				3.00	1.88	3.31		2.46	4.56
	*p*	0.001	0.100				0.001		0.000			0.018					0.058	0.097		0.012		0.011				0.006	0.071	0.003		0.021	0.000
Relief	β													0.28			−0.33		0.32							1.21		−1.09			
	*t*													1.73			1.98		2.16							2.34		2.15			
	*p*												0.092			0.055		0.038							0.025		0.038				
Sadness	β	1.01	−0.53			0.41		−0.21		−0.50	−0.60		−0.42	−0.25									−0.30								−0.28
	*t*	5.01	3.47			2.80		1.75		2.73	4.26		3.20	1.84									2.43								1.82
	*p*	0.000	0.002			0.009		0.090		0.011	0.000		0.003	0.076									0.021								0.079
Contempt	β	0.54	−0.35	0.62		0.27	−1.20		0.50	−0.55					−0.53		0.34			0.95			−0.41			0.31		0.36			−0.43
	*t*	2.37	2.14	3.05		2.08	4.65		3.63	2.34					4.11		2.35			3.64			3.34			2.32		3.06			2.18
	*p*	0.026	0.042	0.005		0.047	0.000		0.001	0.028					0.000		0.027			0.001			0.003			0.029		0.005			0.038
Fear	β	0.23			0.29			−0.31	−0.55				−0.29		0.27	0.35															−0.40
	*t*	1.85			3.03			3.20	5.66				2.79		2.71	3.42															3.36
	*p*	0.074			0.005			0.003	0.000				0.009		0.011	0.002															0.002
Shame	β		0.58	−1.11	0.42		0.69				−1.69	0.71	−0.49	−0.46	−0.36	2.20	−0.85	0.55		2.63	−2.10	1.04	−0.53	−1.00	−7.40		5.56	0.28	−1.14		−0.43
	*t*		2.89	4.34	3.21		1.79				3.71	2.95	3.22	2.56	1.93	5.26	3.63	3.13		4.68	4.65	4.17	2.92	5.52	4.52		4.74	2.37	4.09		1.97
	*p*		0.010	0.000	0.005		0.090				0.002	0.008	0.005	0.020	0.070	0.000	0.002	0.006		0.000	0.000	0.001	0.009	0.000	0.000		0.000	0.029	0.001		0.065
Suddenness	β		−0.47		0.55	0.29								−0.45		0.87				0.37	−0.41	0.42			−1.33		1.17		−0.71		
	*t*		3.22		4.25	2.08								3.08		4.27				1.71	2.26	3.41			2.40		2.42		4.46		
	*p*		0.003		0.000	0.046								0.005		0.000				0.098	0.032	0.002			0.023		0.022		0.000		
Goal obstruction	β		−0.57		0.48			−0.32				−0.21			0.26	0.44															
	*t*		4.19		3.71			2.56				1.70			2.13	3.47															
	*p*		0.000		0.001			0.015				0.098			0.040	0.001															
Relevance and discrepancy	β		−0.63		0.46	0.41								−0.50	0.34	0.56									−0.94	0.24	1.00		−0.49		
	*t*		4.16		3.27	2.62								3.15	2.71	3.10									1.94	1.78	2.12		3.16		
	*p*		0.000		0.003	0.014								0.004	0.011	0.004									0.062	0.085	0.042		0.004		
Coping potential	β	−0.65	−0.61	0.51					−0.29		1.22	−0.72	0.24			−0.40				−1.64	1.05		0.38	0.31	2.80		−1.92		0.44		0.62
	*t*	3.19	3.77	2.57					1.80		4.48	4.13	1.73			1.81				3.71	3.92		2.96	2.50	3.51		3.24		2.59		3.58
	*p*	0.004	0.001	0.017					0.084		0.000	0.000	0.096			0.083				0.001	0.001		0.007	0.020	0.002		0.004		0.016		0.002
External standards violation	β		−0.28				−0.67				0.54			−0.25		0.59										0.28	0.50				−0.48
	*t*		2.12				3.14				3.07			1.70		3.70										1.84	2.46				2.53
	*p*		0.042				0.004				0.004			0.099		0.001										0.075	0.020				0.017
Internal standards violation	β	−0.35	−1.07	0.38	0.27	0.33		−0.53	−0.43		0.97	−0.43		−0.40					−0.21	−1.47	0.98		0.48	0.34	3.56		−2.60				0.31
	*t*	1.94	6.10	2.18	2.14	2.44		2.91	2.90		3.75	2.70		2.96					1.78	3.55	4.45		4.01	3.11	4.09		3.68				1.93
	*p*	0.066	0.000	0.041	0.044	0.023		0.008	0.008		0.001	0.013		0.007					0.090	0.002	0.000		0.001	0.005	0.000		0.001				0.067

At the same time, we found several unexpected positive associations between AUs and recognition of emotions/appraisals. For instance, AU 16 (lower lip depressor) was associated with fear as well as goal obstruction. It is interesting to note that there were also unexpected negative associations between facial actions and emotion/appraisal attribution (see Table 4). For example, the AU12 (smile) had negative associations with the attribution of some negative emotions, such as sadness and shame, but not with any appraisals. In terms of appraisal attribution, a negative association was observed for instance for AU 2 (outer brow raiser) and coping potential.

## Discussion

In our study we looked at the decoding of emotions and cognitive appraisals from sets of AUs seen in a naturally negative emotional setting and we addressed this question through stepwise regression analyses. Results supported our predictions and revealed the relationships between AUs and the decoding of all emotions and cognitive appraisals. These results are consistent with some previous theories postulating the relationships between decoding of emotional categories or cognitive appraisals and sets of AUs (Ekman, 1992; Scherer and Ellgring, 2007), although other theories questioned such relationships (see Barrett et al., 2018). The results are also consistent with previous empirical studies investigating these relationships (e.g., Kohler et al., 2004). However, previous studies did not test spontaneous emotional expressions in naturalistic settings, and hence, the generalizability of these relationships to real-life facial expression processing remained unclear. Extending the current theoretical and empirical knowledge, our results suggest that decoding of emotional categories and cognitive appraisals can be accomplished through the recognition of specific facial movements.

The profiles of AUs associated with the decoding of emotional categories and cognitive appraisals were at least partially consistent with those in previous theories (Ekman, 1992; Scherer and Ellgring, 2007). For instance, the duration of the AU 1 (inner brow raise) and AU 12 (upward lip corner pulling) was associated with the attribution of sadness and joy, respectively. The duration of the AU 4 (brow lowering) and AU 17 (chin raise) was associated with coping potential and goal obstruction, respectively. These findings are also consistent with previous studies with actors (e.g., Kohler et al., 2004). Our results empirically support the notion that these AUs could be the core facial movements to decode emotional categories and cognitive appraisal in natural, spontaneous facial expressions.

At the same time, our results also showed several inconsistent patterns with theoretical predictions (Ekman, 1992; Scherer and Ellgring, 2007). For example, outer brow raiser was not associated with suddenness and lower lip depressor was associated with fear as well as with goal obstruction. Further testing is required for validation purposes in dynamic naturalistic settings as it might be useful to include these AUs in the new theories regarding the relationships between emotion/appraisal decoding and AUs. Furthermore, our results revealed some negative relationships between the duration of AUs and the decoding of emotions/appraisal. This is consistent with results from one rating study of photographs of acted emotional expressions (Galati et al., 1997). In our study, for example, smiles were negatively associated with sadness and shame. These findings suggest that not only the present but also the absent facial movements can be decoded as messages of emotions or appraisals in natural, dynamic, face-to-face communication.

Our findings specifying the relationships between the decoding of emotions/appraisals and AUs in spontaneous facial expressions could have practical implications. For example, it may be possible to build artificial intelligent systems to read emotions/appraisals from emotional facial expressions in a more human-like way. Although such systems currently exist, almost all of them appear to be constructed based on theories or data with actors' deliberate expressions (Paleari et al., 2007; Niewiadomski et al., 2011; Ravikumar et al., 2016; Fourati and Pelachaud, 2018). Additionally, it may be possible to build humanoid virtual agents and robots (Poggi and Pelachaud, 2000; Lim and Okuno, 2015; Niewiadomski and Pelachaud, 2015) for applications in healthcare or in the long term with the elderly, with expressions, which could be recognized as showing natural human-like emotional expressions. Finally, given the importance of appropriate understanding of inner states displayed in others' faces in healthy social functioning (McGlade et al., 2008), it may be interesting to assess the relationships between decoding of emotions/appraisals and AUs using naturalistic facial expression stimuli in clinical conditions. Indeed, several clinical populations report social cognition impairments in real-life situations, while showing satisfactory performance in typical emotion recognition or theory of mind tasks, which mostly rely on the judgment of pictures of acted facial expressions or exaggerated social stories (e.g. see Bala et al., 2018). Dynamic and more naturalistic approaches might help define clinical impairments faced for example by patients with Schizophrenia (Okruszek et al., 2015; Okruszek, 2018), amygdala lesions (Bala et al., 2018) or high functioning autism (Murray et al., 2017) and eventually lead to the improvement of existing social cognition trainings.

Several limitations of the present study should be acknowledged. First, our naturalistic set was limited in the number of stimuli and included only a negatively valenced situation at a single location. In order to generalize the findings, more positive and negative situations presented in varied and controlled contexts and cultures would need to be investigated. Furthermore, although we lacked data regarding emotions experienced by the expressers and we did not monitor the internal states of the participants, it would have been interesting to investigate interactions between AUs and encoded/decoded emotions and the characteristics of the observers. Given the literature on how emotions, facial mimicry and moods of observers influence emotion perception in others (e.g., Schmid and Schmid Mast, 2010; Wood et al., 2016; Wingenbach et al., 2018) it is a valuable topic in future research on dynamic naturalistic stimuli interpretation. Second, we analyzed only male participants. Although consistent gender differences have not been reported in terms of rating-style in the decoding of emotional expressions (e.g., Duhaney and McKelvie, 1993; Biele and Grabowska, 2006; for a review, see Forni-Santos and Osório, 2015), numerous studies have reported that the gender of the decoder might influence different aspects of the processing of faces. For example, the recognition of gender of faces was enhanced (reaction time reduced) when these were presented looking away from the decoder of opposite gender, but not in the case of a same gender decoder (Vuilleumier et al., 2005). It has also been reported that exposure to angry male as opposed to angry female faces activated the visual cortex and the anterior cingulate gyrus significantly more in men than in women (Fischer et al., 2004). Similarly, although no significant differences were observed in accuracy ratings by male vs. female participants nor in the recognition of male vs. female encoder faces, higher brain activity was observed in the extrastriate body area in reaction to threatening male faces compared to female faces, as well as in the activity of the amygdala to threatening vs. neutral female faces in male but not female participants (Kret et al., 2011). For all those reasons, the effect of gender of decoder participants needs to be carefully monitored in further studies. Third, although our final models had high statistical power, our sample size was small. In our approach we used stepwise regression analyses in order to select a subset of predictor variables. While having expected a number of predictor variables to be observed based on previous evidence, and our analyses having detected the expected number of predictor variables with high power, our analyses lacked the power to sufficiently analyse AUs not included in the final models. Future studies with a larger sample size may reveal the involvement of more AUs in the decoding of emotions/appraisals. Fourth, we coded single AUs but not the combination (i.e., simultaneous appearance) of AUs (e.g., AU 6 + 12) due to the lack of power. Because single vs. combined AUs could transmit different emotional messages (Ekman and Friesen, 1975), investigation of AU combination is an important matter for future research. Fifth, we coded AUs in a binary fashion as conducted in the previous studies testing the AUs and decoding of emotions (e.g., Kohler et al., 2004). The coding of 5-level AU intensity, which were newly added in FACS coding (Ekman et al., 2002), may provide more detailed insights regarding the relationships. Sixth, we studied only a linear additive relationship between AUs and the decoding of emotions and their components to simplify analyses. Further work could go beyond linear associations, e.g., quadratic associations. Finally, the use of naturalistic behavior in perceptive paradigms only allows for correlational studies, without the possibility of any strong claims of causality. When constructing paradigms allowing for causality testing, one aspect of interest for future investigations is the direct influence of single facial units on attributions, and future studies could carefully manipulate the presentation of AUs while keeping as much as possible of a naturalistic setting. One method to manipulate behavior one-by-one is to reproduce human behavior using a virtual humanoid or robot. Today's technology allows for dynamic and functional representations of human behavior, which can be copied from a naturalistic scene in sufficient detail in order to evoke similar reactions to the one's observed in videos of humans. Given that presenting behavior without context or one AU at a time lacks *naturalness*, AUs should be judged in sets of units they originally appear in. The manipulation of single AUs could focus on the removal of existing actions (see Hyniewska, 2013).

In conclusion, numerous studies have investigated the decoding of emotional expressions from prototypical displays and there seems to be unanimity on sets of facial AUs that provide good discriminability. However, to the authors' best knowledge, no study has looked at sets of AUs that lead to emotion and appraisal perception in naturally occurring situations. Our results show that emotional and appraisal labels can be predicted based on recorded sets of facial actions units. Interestingly, the sets of observed AUs do not coincide with what has been observed in former decoding studies.

## Author Contributions

SH, SK, and CP were responsible for the conception and design of the study. SH obtained the data. SH and WS analyzed the data. All authors wrote the manuscript.

### Conflict of Interest Statement

The authors declare that the research was conducted in the absence of any commercial or financial relationships that could be construed as a potential conflict of interest.
