# Supplementary material for: Naturalistic Emotion Decoding From Facial Action Sets
Source: Front Psychol. 2019 Jan 18;9:2678. doi: 10.3389/fpsyg.2018.02678 (PMC6345715; doi:10.3389/fpsyg.2018.02678)
Supplement: Supplementary file 1 [file Data_Sheet_1.PDF]

Table A1. Theoretical associations between emotional categories and action units.

Emotion	Action unit
Joy	1, 2, (5), 6 + 12, 26
Anger	1, 2, 4, 5, 6, 7, 10, 23, (24), 25, 26, 27
Sadness	1, 4, 5, 15, 17, 25
Contempt	4, 7, 10, 11, 15, 17, 25, 26
Fear	1, 2, 4, 5, (7), 20, 25
Shame	1, 2, 4, 5, 14, 22, 23, 25

Derived from Scherer (2001) and Ekman's Emotional dictionary — available on the FACSAID CD (Ekman et al., 1998). "+" signifies simultaneous AU and "," signifies alternative ones.

Table A2. Theoretical associations between cognitive appraisals and action units.

Cognitive appraisal	Action unit
Suddenness	1 + 2
Goal obstruction	17 + 23; 17 + 24
Relevance and discrepancy	4, 7, 23, 17, gaze directed
Coping potential	4, 5, 7, 17+23, 24, 25
External standards violation	14 and no 10
Internal standards violation	14

Derived from Scherer and Ellgring (2007), Kaiser and Wehrle (2001), and Alvarado & Jameson (2002). "+" signifies simultaneous AU and "," signifies alternative ones.
